# CircTHSD4 promotes the malignancy and docetaxel (DTX) resistance in prostate cancer by regulating miR-203/HMGA2 axis

**DOI:** 10.32604/or.2023.031511

**Published:** 2024-02-06

**Authors:** JIANYUN XIE, LINJIE LU, JIALI ZHANG, QIRUI LI, WEIDONG CHEN

**Affiliations:** 1 Department of Urology, The Affiliated People’s Hospital of Fujian University of Traditional Chinese Medicine, Fuzhou, China; 2 Department of Internal Medicine Outpatient Clinic, The Affiliated People’s Hospital of Fujian University of Traditional Chinese Medicine, Fuzhou, China

**Keywords:** circTHSD4, Docetaxel resistance, Prostate cancer, miR-203, HMGA2

## Abstract

**Objective:**

Circular ribose nucleic acids (circRNAs) are implicated in tumor progression and drug resistance of prostate cancer (PCa). The current work explored the function of circ_0005203 (circTHSD4) in the malignancy and docetaxel (DTX) resistance of PCa.

**Methods:**

circTHSD4 expression within PCa as well as matched non-carcinoma samples was measured through real-time reverse transcription-quantitative polymerase chain reaction (RT-qPCR). In addition, a subcellular fraction assay was conducted to determine circTHSD4 subcellular localization within PCa cells. In addition, we performed a Western blot (WB) assay to detect high-mobility-group A2 protein (HMGA2) levels. Besides, functional associations of two molecules were investigated through dual luciferase reporter assay. Cell Counting Kit (CCK)-8, colony formation together with Transwell assay was conducted to assess malignant phenotypes of PCa cells, whereas flow cytometry was performed to determine cell apoptosis. Furthermore, a xenograft mouse model was constructed to verify the effect of circTHSD4 on the carcinogenesis of PCa cells.

**Results:**

According to RT-qPCR results, circTHSD4 was up-regulated within PCa tissues and cells, which predicted the dismal prognostic outcome of PCa cases. circTHSD4 silencing within PCa cells markedly suppressed cell growth, migration, and colony formation. circTHSD4 silencing remarkably elevated PCa cell apoptosis and carcinogenesis within the xenograft model. Further, circTHSD4 silencing enhanced docetaxel (DTX) sensitivity in PCa cells. Furthermore, we demonstrated that circTHSD4 modulated the malignancy of PCa cells by regulating HMGA2 expression through sponging miR-203.

**Conclusion:**

Together, our findings suggest that circTHSD4 overexpression could promote the malignant phenotype and DTX resistance in PCa through the regulation of the miR-203/HMGA2 axis.

## Introduction

Prostate cancer (PCa) refers to the most commonly diagnosed malignancy in male genitourinary system, ranking as the fifth major reason for cancer-related deaths [1]. In accordance with the epidemiological data among 36 different cancers in 185 different nations, there were 375,304 (3.8%) fatalities and 1,414,259 (7.3%) newly diagnosed cases of PCa in 2020 [2]. Currently, a combination of surgery with adjuvant radiotherapy and chemotherapy remains as the mainstay therapy for PCa patients. Nevertheless, the development of multidrug resistance (MDR) is a pervasive issue in the clinical management of PCa, which has been recognized as the major reason for treatment failure and the mortality in PCa patients [3].

Endocrine therapy represents a standard treatment for PCa patients. However, PCa frequently progresses or relapses into androgen-independent prostate cancer (AIPC) and hormone-refractory prostate cancer (HRPC) after continuous endocrine therapy [4]. Docetaxel (DTX) is one of the most frequently used anticancer drugs for PCa treatment. The standard chemotherapeutic regimen of DTX can significantly improve the survival of PCa patients. However, the development of DTX resistance seems to be an inevitable consequence of PCa chemotherapy, and the median survival duration of drug-resistant patients is as low as 18 months [4]. As a result, the investigation of molecular mechanisms underlying DTX resistance could offer novel insights into the formulation of novel strategies to target DTX drug resistance.

Circular ribose nucleic acids (circRNAs) are a class of closed-loop RNAs that are usually produced by reverse splicing of the exons or introns [5]. CircRNAs are predominantly accumulated in the cytoplasm or exosomes and are highly conserved across species but expressed in a tissue-dependent manner [6]. Recently, circRNAs have emerged as critical regulators in a variety of biological processes and form complex regulatory networks with other non-coding RNAs [7]. Several oncological studies reported that circRNAs played crucial roles in tumor progression and the development of chemotherapeutic resistance [8,9]. Besides, the deregulation of circRNAs is also implicated in the diagnosis and prognosis of PCa [10]. For instance, circABCC4 was reported as a competitive endogenous RNA (ceRNA) of miR-1182 and its overexpression promotes the growth of PCa cells by upregulating Forkhead Box P4 (FOXP4) expression [11]. In another study, circHIPK3 was discovered to sponge miR-338-3p and promote the G2/M transition of PCa cells [12]. It was previously discovered that circRNA Foxo3 was downregulated in PCa and its reduced expression contributed to the development of resistance to DTX [13]. In another similar study, circ-XIAP was secreted by exosome functions to foster the resistance of PCa cells to doxorubicin via the regulation of the miR-1182/Tumor Protein D52 (TPD52) axis [14].

High Mobility Group AT-Hook 2 (HMGA2) is a member of the high mobility group protein family [15]. HMGA2 interacts with the AT-rich regions of DNA through the AT-hook domain to regulate DNA structure [16]. Although HMGA2 does not possess intrinsic transcriptional activity, HMGA2 forms a transcription factor complex to regulate gene expression through the interaction with the chromatin. The altered transcription profiles due to HMGA2 expression changes can regulate embryonic development, cell differentiation and apoptosis [17]. Previous studies have implicated HMGA2 in the differentiation, transformation, proliferation, and metastasis of malignant cells of different cancers [18–20].

A previous study reported the overexpression of hsa_circ_0005203 (circTHSD4) in PCa by circRNA sequencing analysis [21]. Nevertheless, the specific role of circTHSD4 in the tumor progression and drug resistance of PCa remains to be explored. The current work focused on reporting that circTHSD4 expression was dramatically upregulated in PCa tissues, which was associated with the downregulation of the tumor suppressor factor miR-203 in PCa [22]. We further demonstrated that miR-203 binds to the 3′ untranslated region (UTR) of HMGA2 mRNA. The obtained findings showed that circTHSD4 exerts a vital role in promoting the malignancy and DTX resistance in PCa by regulating miR-203/HMGA2 axis.

## Materials and Methods

### Bioinformatics prediction

The CircInteractome (https://circinteractome.nia.nih.gov/), a circRNA online bioinformatics tool, was adopted for the prediction of the target miRNAs of hsa_circ_0005203 (circTHSD4). The TargetScan (v7.0) (http://www.targetscan.org/vert_72/docs/help.html), miRDB (http://mirdb.org/), and starBase (v2.0) (https://rnasysu.com/encori/index.php) databases were employed to identify potential mRNA candidates of miR-203.

### Clinical samples

The tumor samples and paracancerous specimens from the PCa patients (n = 88) were gathered at The Affiliated People’s Hospital of Fujian University of Traditional Chinese Medicine between June-2016 and August-2022. Those included subjects received surgery with no prior chemotherapy or radiotherapy. Tissue samples collected were immersed in liquid nitrogen for snap-freezing under −196°C. Every participant provided informed consent before participating in the present work. All clinical samples were used following guidelines of the Declaration of Helsinki (Seventh Revision) [23]. The Ethics Committee of the Affiliated People’s Hospital of Fujian University of Traditional Chinese Medicine (20160329) approved our experimental protocols on human tissues.

### Cell culture

This work acquired healthy human prostate epithelial cells (RWPE-1) along with diverse PCa cells (PC3, DU145, VCaP, and LNCaP) from Typical Culture Repository of China, Institute of Science (Shanghai, China) and cultured them within Roswell Park Memorial Institute (RPMI)-1640 cell medium that contained 10% fetal bovine serum (12662011, Gibco, Waltham, WI, USA) under 37°C with 5% CO_2_.

### Subcellular localization of circRNA

The cytoplasmic and nuclear fractions of PCa cells were extracted with the use of the Cytoplasmic & Nuclear RNA Purification Kit (NGB-21000, NorgenBiotek, Thorold, Ontario, Canada). The total RNA sample from each fraction was purified by the Trizol reagent (L28164, Beyotime, Beijing, China). The RNA samples were then exposed to RT-qPCR analysis to detect circTHSD4 and THSD4 mRNA levels.

### Cell transfection

The sequences of circTHSD4 shRNA (sh-circTHSD4 1#, 2#, 3#) and negative control (pLKO.1-scramble shRNA), pcDNA3.1-HMGA2 overexpression vector, and the control vector (pcDNA3.1-vector) were purchased from GenePharma (Shanghai, China). Besides, Ribobio (Guangzhou, China) was responsible for preparing negative control (NC) oligonucleotides, has-miR-203 mimics, and hsa-miR-203 inhibitors. Thereafter, PC3 and DU145 cells (1 × 10^7^/well) were inoculated into 6-well plates and later transfected with oligonucleotides and corresponding vectors with Lipofectamine® 3000 reagent (L3000001, Invitrogen, Carlsbad, CA, USA). For generating cells showing stable circTHSD4 silencing, 2 g/mL puromycin was added to screen PC3 and DU145 cells for a 2-week period. At 48-h post-transfection, RT-qPCR assay was carried out to identify transfection efficiency. The target sequences for circTHSD4 shRNA are as follow: 1# GCAAGTATGGCTATGGTAAGG, 2# GCCCAGAAACAAGCAACAACC, and 3# GCATTGGCTGTGATGACTACT.

### Cell proliferation assay

The proliferation of cells was identified by the cell counting kit (CCK)-8 assay. In brief, PC3 and DU145 cells in the logarithmic growth phase were seeded in the 96-well plate at the density of 1 × 10^4^ cells/well. The cells were subject to incubation for 0, 24, 48, and 72 h, respectively. In the meanwhile, 10 μl of CCK-8 reagent (C0039, Beyotime, Beijing, China) was supplemented to each well for 3 h incubation. The absorbance of each well was determined with the application of iMark™ Microplate Absorbance Reader (Bio-Rad, CA, USA) at an OD_450_ nm.

### Colony formation assay

PC3 and DU145 cells at the logarithmic phase were collected and prepared into suspensions at 1 × 10^3^ cells/well in the 6-well plate. Growth medium was discarded following 2-week culture, followed by addition of 4% paraformaldehyde to achieve 30-min cell fixation and introduction of 0.1% crystal violet (C0121, Beyotime, Beijing, China) to achieve 20-min cell staining. Colony number was determined with the light microscope (magnification, 20×). The formula below was adopted to identify clonogenic rate: clonogenic rate (%) = (clone number/injected cell number) × 100.

### Transwell experiment

The migration and invasion abilities were identified using the transwell assay. The serum-free culture medium was applied to dilute Matrigel (354230, BD Biosciences, CA, USA), and the bottom of the top chamber in the Transwell plates (3422, Corning, Beijing, China) was coated with the Matrigel for the invasion assay. The migration assay was carried out in the Transwell plate without Matrigel coating. Totally 5 × 10^4^ cells were seeded in the upper chamber using serum-free culture medium. The lower chamber was supplemented with the medium with 20% FBS. After a day, the cells on the membrane were fixed with 4% paraformaldehyde for 10 min and later stained for 20 min with 0.1% crystal violet solution (C0121, Beyotime, Beijing, China). For each sample, three randomly chosen microscopic fields were used for quantification under an inverted microscope (magnification of 100×).

### Flow cytometric detection of apoptosis

Cell apoptosis was determined using Annexin-V-fluorescein isothiocyanate (FITC) cell apoptosis detection kit (K201-100, BioVision, Palo Alto, CA, USA). The 1× binding buffer that contained Annexin-V-FITC as well as propidium iodide (PI) was added for cell resuspension at 1 × 10^6^/ml and another 15-min staining in dark. After washing by washing buffer twice, binding buffer (500 μL) was added for cell resuspension. Apoptotic cell rate was measured by using BD FACS CantoTM II Flow Cytometer (BD Biosciences, CA, USA).

### Biotinylated miRNA pull-down test

The biotinylated miRNA mimics (hsa-miR-1197, hsa-miR-203, hsa-miR-1252-5p, hsa-miR-615-5p, hsa-miR-1322, and hsa-miR-637) or the control miR-NC (RiboBio, Guangzhou, China) were initially transfected into PC3 and DU145 cells. IP lysis buffer (P0013, Beyotime, Beijing, China) was used to collect cell lysates at 2 days later, which were later subjected to overnight incubation using M-280 streptavidin magnetic beads (Sigma-Aldrich, 11205D, Shanghai, China) under 4°C. Magnetic beads together with relevant nucleic acids were pulled down with the magnetic bar. The RNAs from the input and the pull-down samples were purified with Trizol reagent (L28164, Beyotime, Beijing, China) in accordance with the instruction of the manufacturer. Finally, the relative amount of enriched circTHSD4 was measured by RT-qPCR analysis.

### Dual-luciferase assay

Initially, the potential binding sites between circTHSD4/miR-203 and miR-203/HMGA2 mRNA were predicted by employing starBase (v2.0) and TargetScan (v7.2) databases. The wild type (WT) or mutated (MUT) binding sites were cloned into the pmirGLO vector using a TOPO TA cloning kit (452640, Invitrogen, Shanghai, China). Using the Lipofectamine® 3000 reagent, the co-transfection of miR-203 mimics or miR-NC and the WT or MUT reporters into the PC3 and DU145 cells was conducted. 48 h post-transfection, the relative luciferase activities were determined with a dual-luciferase reporter assay kit (E1910, Progema, WI, USA) based on the manufacturer's instructions.

### RNA immunoprecipitation (RIP) assay

IP lysis buffer (P0013, Beyotime, Beijing, China) was employed for cell lysis. Thereafter, Magna RIP RNA-Binding Protein Immunoprecipitation Kit (17-700, Millipore, CA, USA) was utilized to incubate cells in RIP assay. Then, the rabbit anti-Ago2 antibody (ab32381) or negative control normal rabbit anti-IgG (ab188776, Abcam, Cambridge, UK) was conjugated onto kits-derived protein A/G magnetic beads. Later, cell lysates were incubated using the resultant antibody-bead mixture overnight under 4°C. The magnetic bar was adopted for the precipitation of magnetic beads. Then, samples were washed by NT2 buffer twice. The eluted samples were purified with Trizol reagent (L28164, Beyotime, Beijing, China), and RT-qPCR analysis was adopted for quantifying the relative enrichment of each molecule.

### RT-qPCR assay

with the use of the TRIzol reagent The total RNA samples from tissues and cells were extracted (L28164, Beyotime, Beijing, China). The total RNA was reverse transcribed into cDNA with the One-Step PrimeScript miRNA cDNA kit or PrimeScript RT kit (RR037B, Takara, Kusatsu, Japan). Further, the cDNAs were amplified in the qPCR reaction with the SYBR-Green Master Mix kit (RR820A, Takara, Kusatsu, Japan) on the ABI7500 qPCR system (ABI, CA USA). The relative expressions of target genes were identified with the 2^−ΔΔCt^ method. GAPDH was applied as the internal reference for protein-coding gene and the circRNA derived from the protein-coding sequence, whereas snRNA U6 was used to be its internal reference gene for miR-203 quantification. The primers are presented in Suppl. Table S1 in the supplementary information.

### RNase R treatment

RNase R was applied to degrade linear RNA. The RNA sample was divided equally into two portions including one was adopted for Rnase R digestion (Rnase R+ group), and the other was applied control (Rnase R- group). 2 μg of total RNA was treated with or without 3 U/μg RNase R (ISO-13485, Lucigen, WI, USA) at 37°C for 15 min. Further, RT-qPCR analysis was adopted for identifying the relative amount of THSD4 and circTHSD4 before and after the treatment.

### Western blot (WB) assay

PC3 and DU145 cells were collected during the logarithmic growth phase and lysed on ice using the RIPA lysis solution including protease inhibitors (P00013B, Beyotime, Beijing, China). A BCA protein assay kit (P0006, Beyotime, Beijing, China) was adopted for quantifying the overall protein content. Subsequently, 50 μg of total protein was subject to 10% sodium dodecyl-sulfate polyacrylamide gel electrophoresis (SDS-PAGE). The separated proteins were transferred to a polyvinylidene difluoride membrane (IPVH00010 Millipore, MA, USA). The membranes were blocked with 5% skim milk at room temperature for 60 min and washed thrice with Tris-buffered saline with Tween-20 (TBST). Then, the blots were subject to incubation overnight at 4°C with primary antibodies: anti-HMGA2 antibody (PA5-114731, 1:1000; Invitrogen, CA, USA) and anti-GAPDH (ab8245, 1:2000; Abcam, Cambridge, UK). After washing using TBST buffer, the membrane was further subject to incubation with a goat anti-rabbit secondary antibody linked with horseradish peroxidase (HRP, ab288151, 1:5000; Abcam) at room temperature for 1 h. In addition, the signal development was performed with an enhanced Chemiluminescence (ECL) kit (P0018FS, Beyotime, Beijing, China) and the protein bands were explored with Image J. (National Institutes of Health, Bethesda, MD, USA).

### Subcutaneous tumorigenesis assay

BALB/c nude mice (female, 4–6 weeks old, 15 g) were purchased from Shanghai Laboratory Animal Center (SLAC, Shanghai, China) and raised in a standard animal facility at 25°C and 12:12-h light cycle. After one week adaptation, the nude mice were subject to injection with 5 × 10^6^ PC-3 cells stably transfected with sh-NC or sh-circTHSD4 at the left flanks (n = 5 per group). The subcutaneous tumor size was measured every a week for 4 weeks. The mice were euthanized by CO_2_ asphyxiation and cervical dislocation on day 28, and the xenograft tumors were gathered for performing further analysis. The animal study was conducted following the ARRIVE guidelines [24] and the experimental protocols were approved by the Animal Care Committee of the Affiliated People’s Hospital of Fujian University of Traditional Chinese Medicine (2021-033). The study also complied with the instruction of National Institutes of Health standards for humane animal care [25].

### Immunohistochemical (IHC) staining

The tumor tissues were preserved in 4% paraformaldehyde for 36 h, and later dehydrated and embedded in paraffin. The tissue block was cut into 4 µm ultra-thin sections. After de-paraffinization and re-hydration, antigen retrieval was performed in Tris/EDTA (pH-8.0 for p16ink4a and pH-6.0 for Ki-67) at sub-boiling temperature 95°C for 5 min. Subsequently, the sections were subject to incubation with anti-Ki67 antibody (ab92742, Abcam; 1:500) overnight at 4°C. After washing using TBST buffer, the sections were further subject to incubation with a secondary antibody (HRP anti-rabbit IgG antibody, ab288151, Abcam, 1; 2000) for 2 h at room temperature. Color development was conducted using the AB Detection IHC Detection Kit (ab64238, Abcam). Then the sections were dehydrated in 70% ethanol/0.1% hydrochloric acid for 10 s before washing with the distilled water. Using a phase-contrast microscope (DMI6000B; Leica, Wetzlar, Germany), the images were captured.

### Statistical analysis

The experimental data were indicated to be mean and standard deviations (x ± s), and data analysis was statistically explored with the use of SPSS 23.0 (SPSS, Chicago, IL, USA). The comparisons between two groups were explored using the Student’s *t*-test, and comparisons between multiple groups were investigated by one-way analysis of variance (ANOVA) and the *post-hoc* Bonferroni test. For the comparison of expression data in the PCa tumors and para-cancerous specimens, Wilcoxon test was used for determining the similarity of the distributions. The overall survival rate of PCa patients was explored based on the Kaplan-Meier (KM) curve and the log-rank test. A difference with a *p*-value of less than 0.05 was thought to be of statistical significance.

## Results

### circTHSD4 is overexpressed in the PCa tissues and cells

**Figure 1:**
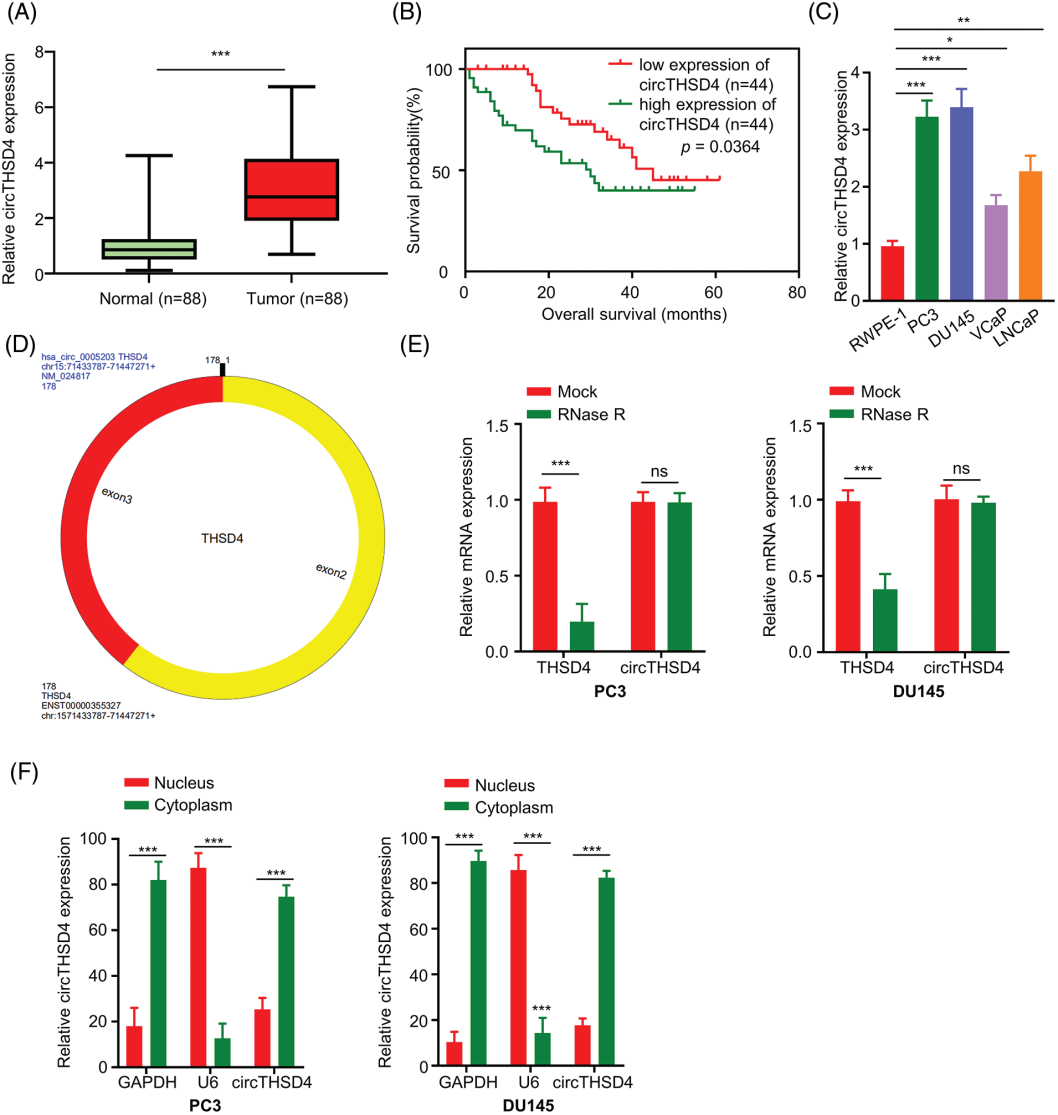
circTHSD4 shows up-regulation within PCa tissues and cells. (A) RT-qPCR assay on circTHSD4 expression in 88 PCa together with 88 matched para-cancerous specimens. (B) The overall survival of patients with high- or low-circTHSD4 expression detected by Kaplan-Meier curves. (C) RT-qPCR analysis on circTHSD4 expression within PCa cells (PC3, DU145, VCaP, LNCaP) as well as a healthy human prostate epithelial cells (RWPE-1). (D) The schematics of circTHSD4 sequence structure. (E) RT-qPCR assay on THSD4 mRNA and circTHSD4 levels within RNase R-treated or non-treated PC3 and DU145 cells. (F) Relative circTHSD4 expression within nucleus and cytoplasm detected by RT-qPCR assay. *p < 0.05, **p < 0.01, ***p < 0.001, and ****p < 0.0001.

We initially quantified the expression levels of circTHSD4 in the 88 pairs of PCa cancer tissues and para-cancerous specimens. It was observed that circTHSD4 showed a significantly higher expression level (*p* < 0.001) in PCa tumor tissues (3.07 ± 1.43) when compared to the normal specimens (0.95 ± 0.68) (Fig. 1A). PCa patients were separated into high (n = 44) and low (n = 44) expression groups in accordance with the median value of circTHSD4 expression in the tumor samples determined by qRT-PCR (Min = 0.70, Max = 6.74, 25% Percentiles = 1.91, 50% Percentiles = 2.77%, and 75% Percentiles = 4.14). The patients with high circTHSD4 levels showed a decreased survival rate than those with low circTHSD4 levels (Fig. 1B). The Chi-square analysis of clinicopathological data and circTHSD4 expression levels revealed significant correlations between the circTHSD4 levels and stage tumor (*p* = 0.010), lymph node metastasis (*p* = 0.001), Gleason score (*p* < 0.001), PSA level (*p* = 0.041), and tumor metastasis (*p* = 0.049) (Table 1). By contrast, no significant relationship was found in relation with patient age (*p* = 0.852), rectal exam (*p* = 0.665), the number of biopsies containing cancer (*p* = 0.153), the percentage of cancer making up each biopsy core sample (%) (*p* = 0.785), and cancer location (*p* = 0.667). Further, circTHSD4 also displayed a significant upregulation in the PCa cell lines (PC3, DU145, VCaP, and LNCap cells) in relative to normal kidney epithelial cells (RWPE-1 cell line) (Fig. 1C). Among various PCa cell lines, PC3 and DU145 cells displayed the highest circTHSD4 expression levels, which were used for further investigations.

**Table 1:** The association between circTHSD4 expression and clinical features in PCa cases

Features	Number	High (n = 44)	Low (n = 44)	t/Χ^2^ value	*p*-value
**Age (years)**	88	61.16 ± 12.76	60.61 ± 14.57	0.187	0.852
**Gleason score**	88	7.50 ± 0.95	4.77 ± 1.55	9.930	<0.001***
**Tumor stage**				6.669	0.010
T1–T2	38	13	25		
T3–T4	50	31	19		
**Lymph-node metastasis**				11.733	0.001
Yes	40	28	12		
No	48	16	32		
**PSA level (ng/ml)**	88	55.64 ± 28.36	43.18 ± 28.04	2.071	0.041
**Rectal exam**				0.188	0.665
Abnormal	52	27	25		
Normal	36	17	19		
**Number of biopsies containing cancer** **△**				2.045	0.153
**Positive (+)**	603	313	290		
**Negative (−)**	453	215	238		
**The percentage of cancer making up each biopsy core sample (%)** ^**◊**^	603	41 ± 14.75	42 ± 18.42	−0.271	0.785
**Location of the cancer**				0.185	0.667
One side	38	18	20		
Both sides	50	26	24		
**Metastasis**				3.879	0.049
Yes	22	15	7		
No	66	29	37		

Note: * indicates *p* < 0.05, ** presents *p* < 0.01, *** defines *p* < 0.001, and **** signifies *p* < 0.0001.

**△** A 12-point puncture pattern was performed for each patient.

^**◊**^ only the samples with positive biopsies were analyzed for the percentage of cancer cells.

circRNA database circRNADb (http://reprod.njmu.edu.cn/cgi-bin/circrnadb/circRNADb.php) was used to derive the structure of circTHSD4. circTHSD4 was formed by the splicing of two exons of THSD4 gene (Fig. 1D). With the aim of validating the circular structure of circTHSD4, RNase R was used to perform linear RNA digestion. After RNase R treatment, THSD4 mRNA level was heavily reduced while circTHSD4 expression level remained unaffected (Fig. 1E). We also collected the nuclear and cytoplasmic fraction of the PCa cells and examined the relative abundance of the circTHSD4. qRT-PCR data demonstrated that circTHSD4 was primarily localized in the cytoplasm of PC3 and DU145 cells (Fig. 1F).

### Silencing circTHSD4 inhibits the proliferation, migration, and invasion of PCa cells

**Figure 2:**
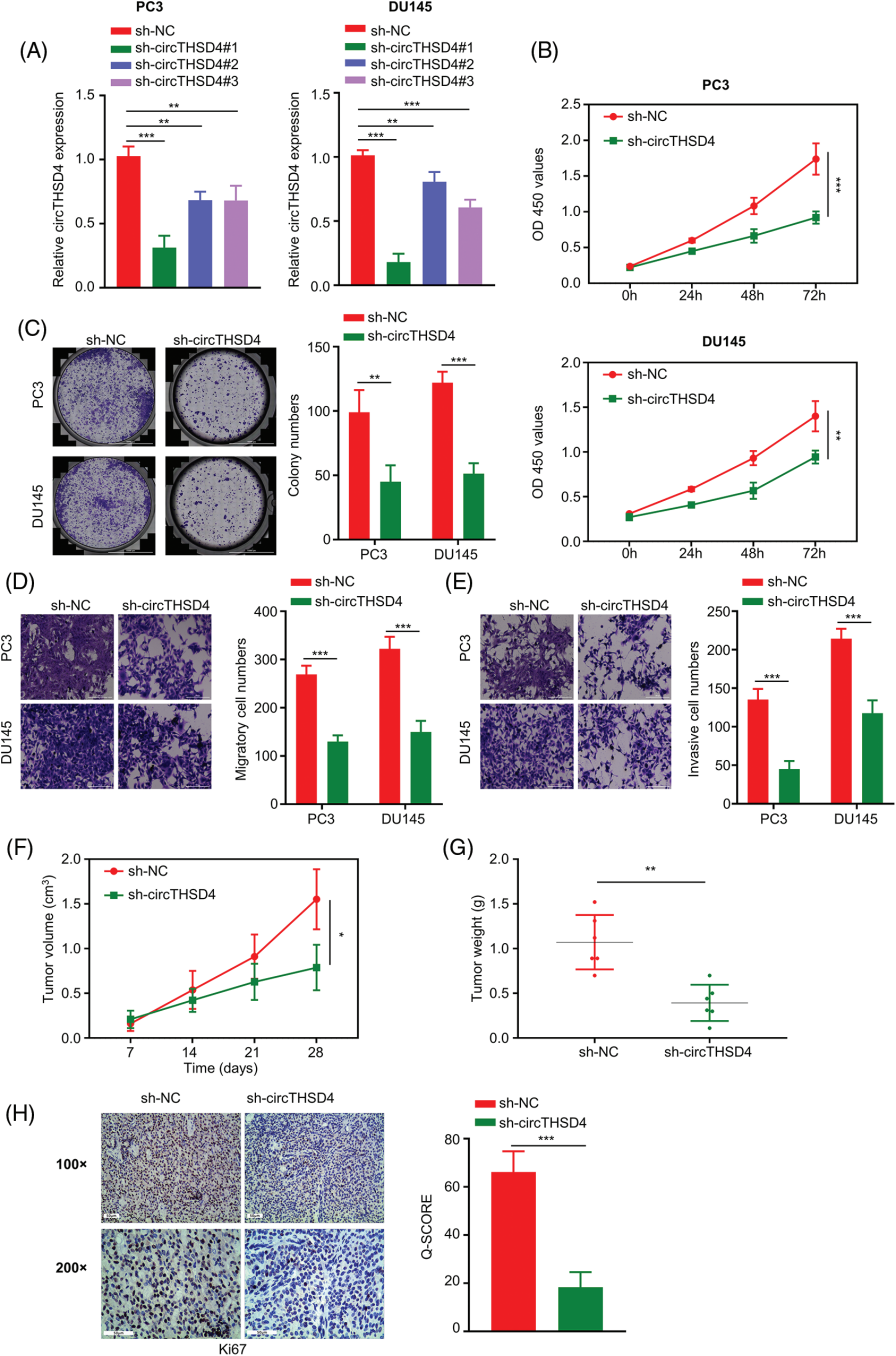
circTHSD4 knockdown inhibits PCa cell growth, invasion and migration. (A) RT-qPCR assay on circTHSD4 levels after lentiviral infection of sh-NC (control shRNA) and sh-circTHSD4 (shRNAs targeting circTHSD4). (B) CCK-8, (C) The colony formation, and (D and E) Transwell assays on PC3 and DU145 cells with sh-NC and sh-circTHSD4 expression. (F) Tumor volume measurement in nude mice given injection of PC3 cells carrying sh-NC and sh-circTHSD4. (G) The summary of xenograft tumor weight on day 28 in sh-NC and sh-circTHSD groups. (H) IHC analysis on Ki-67 staining within cancer sections of sh-NC and sh-circTHSD group. *p < 0.05, **p < 0.01, ***p < 0.001, and ****p < 0.0001.

In order to investigate the biological function of circTHSD4 in PCa cells, lentivirus with shRNA targeting circTHSD4 was used to knock down circTHSD4 in PC3 and DU145 cell lines. In comparison to control shRNA (sh-NC), all three circTHSD4 shRNAs (sh-circTHSD4#1, sh-circTHSD4#2, and sh-circTHSD4#3) could efficiently decrease circTHSD4 expression (*p* < 0.05, Fig. 2A). sh-circTHSD4#1 was used in the subsequent analysis since it showed the greatest knockdown effect. Further, we carried out different functional assays to evaluate the role of circTHSD4 in PCa cells, such as *in vitro* CCK-8, colony formation, Transwell assays, and *in vivo* subcutaneous tumor formation experiment in nude mice. CCK-8 assay results showed the reduced cell proliferation after the knockdown of circTHSD4 (*p* < 0.01, Fig. 2B). Similarly, in the colony formation experiment, circTHSD4 knockdown significantly impaired the colony formation of PCa cells (*p* < 0.01, Fig. 2C). Further, the migration and invasion abilities of PCa cell lines were also inhibited after circTHSD4 knockdown (*p* < 0.01, Figs. 2D and 2E). In the nude mouse model of xenograft, circTHSD4 knockdown also substantially hindered the tumor growth (*p* < 0.01, Figs. 2F and 2G). Consistently, IHC staining of Ki-67 in the subcutaneous tumor tissues revealed that the knockdown of circTHSD4 attenuated Ki-67 expression in the subcutaneous tumor tissues (*p* < 0.01, Fig. 2H). Together, the obtained findings suggest that circTHSD4 acts as a tumor-promoting factor in PCa.

### Increased sensitivity of PCa cells to DTX after circTHSD4 knockdown

**Figure 3:**
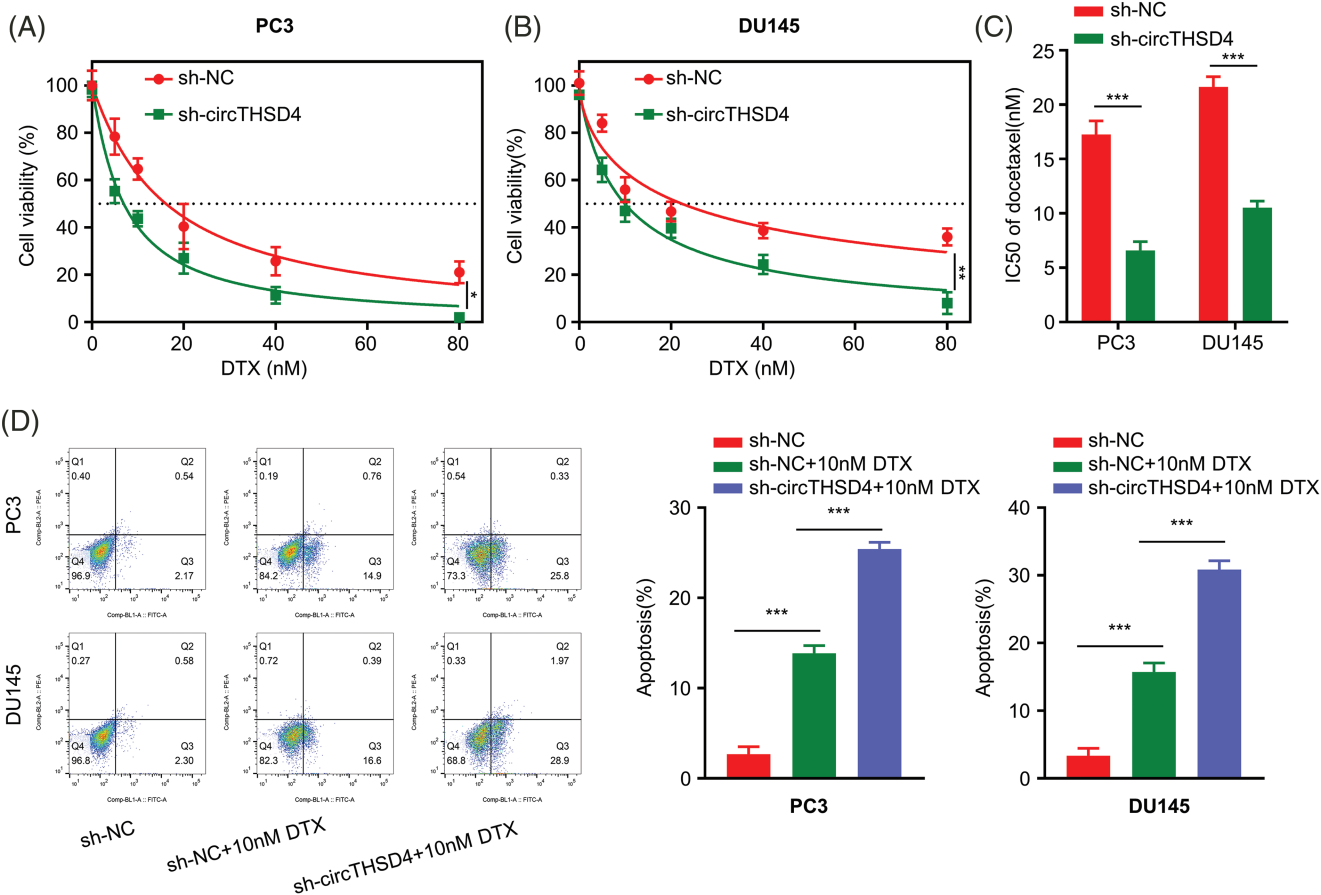
circTHSD4 silencing promotes DTX sensitivity in PCa cells. (A and B) CCK-8 cell viability test of PC3 and DU145 cells following treatment with DTX at various doses for 48 h. (C) The summary of IC50 values within PC3 and DU145 cells with/without circTHSD4 knockdown. (D) Flow cytometry assay of apoptosis cell proportion following DTX administration into PC3 and DU145 cells with/without circTHSD4 knockdown. *p < 0.05, **p < 0.01, ***p < 0.001, and ****p < 0.0001.

Next, we explored the impact of circTHSD4 silencing on the drug sensitivity to DTX treatment. PC3 and DU145 cells (sh-NC and sh-circTHSD4 group) were treated with the elevating doses of DTX (0, 5, 10, 20, 40, and 80 nM) for 48 h and the cell viability was evaluated using CCK-8 assay. Cell viability decreased with the increasing DTX concentration. Meanwhile, sh-circTHSD4 group was more sensitive to DTX treatment than the sh-NC group (*p* < 0.01, Figs. 3A and 3B), which was also manifested as the lower IC50 value (concentration with 50% inhibition) in the sh-circTHSD4 group (*p* < 0.01, Fig. 3C). Moreover, the flow cytometric analyses demonstrated that the apoptotic events were significantly increased upon circTHSD4 knockdown with DTX treatment in both cell lines (*p* < 0.01, Fig. 3D). Thus, silencing circTHSD4 promotes the drug sensitivity of PCa cells to DTX treatment.

### circTHSD4 negatively targets miR-203

**Figure 4:**
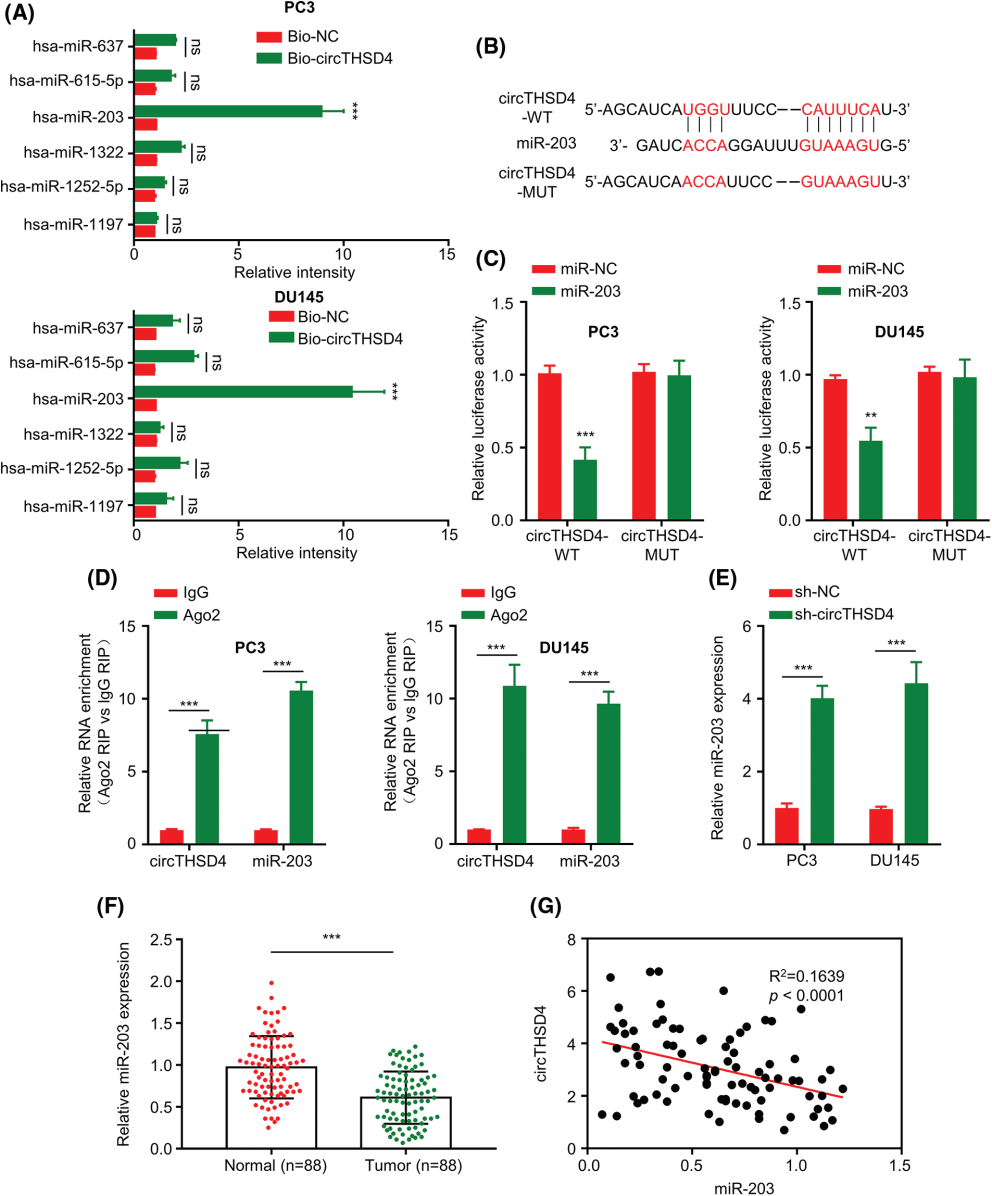
circTHSD4 targets miR-203 within PCa cells. (A) Relative enrichment of miRNA candidates within PC3 and DU145 cells using circTHSD4 or control probe. (B) The schematics of the WT binding site and MUT sequence of circTHSD4 and miR-203. (C) The dual-luciferase reporter analysis of the WT and MUT reporter. (D). RIP-RT-qPCR assay on association between circTHSD4 as well as miR-203 and Ago2 complex. (E) RT-qPCR assay on miR-203 expression within both PC3 and DU145 cell lines following circTHSD4 knockdown. (F) RT-qPCR assay on miR-203 levels within 88 PCa tumor samples together with matched para-cancerous specimens. (G) Relationship of circTHSD4 with miR-203 expression within PCa tumor samples. *p < 0.05, **p < 0.01, ***p < 0.001, and ****p < 0.0001.

The mechanistic action of circTHSD4 was further investigated by exploring the miRNA targets. A total of 6 high-score miRNA candidates of circTHSD4 (hsa-miR-1197, hsa-miR-615-5p, hsa-miR-1252-5p, hsa-miR-637, hsa-miR-1322, and hsa-miR-203) were predicted by circInteractome database. RNA pull-down analysis using Bio-NC and Bio-circTHSD4 probe showed that circTHSD4 could only significantly enrich miR-203 (*p* < 0.01, Fig. 4A). The putative interacting sites between circTHSD4 and miR-203, and the mutated binning sites were displayed in Fig. 4B. Through the luciferase reporter assay, this study demonstrated hat the overexpression of miR-203 notably hindered the WT reporter activity in PCa cells, while the inhibitory effect was abolished after the mutation of the putative binding site in the MUT reporter (Fig. 4C). The association of circTHSD4 and miR-203 was also verified by RIP-RT-qPCR analysis, in which the Ago2 antibody was able to precipitate both circTHSD4 and miR-203 (*p* < 0.01, Fig. 4D). Additionally, miR-203 level was markedly elevated in PC3 and DU145 cells after the knockdown of circTHSD4 (*p* < 0.01, Fig. 4E). There was a clear downregulation of miR-203 expression in the PCa tissues in relative to the para-cancerous tissues (*p* < 0.01, Fig. 4F), and the levels of circTHSD4 and miR-203 displayed an obvious trend of negative correlation (R^2^ = 0.1639, *p* < 0.0001, Fig. 4G).

### HMGA2 mRNA is negatively regulated by miR-203

**Figure 5:**
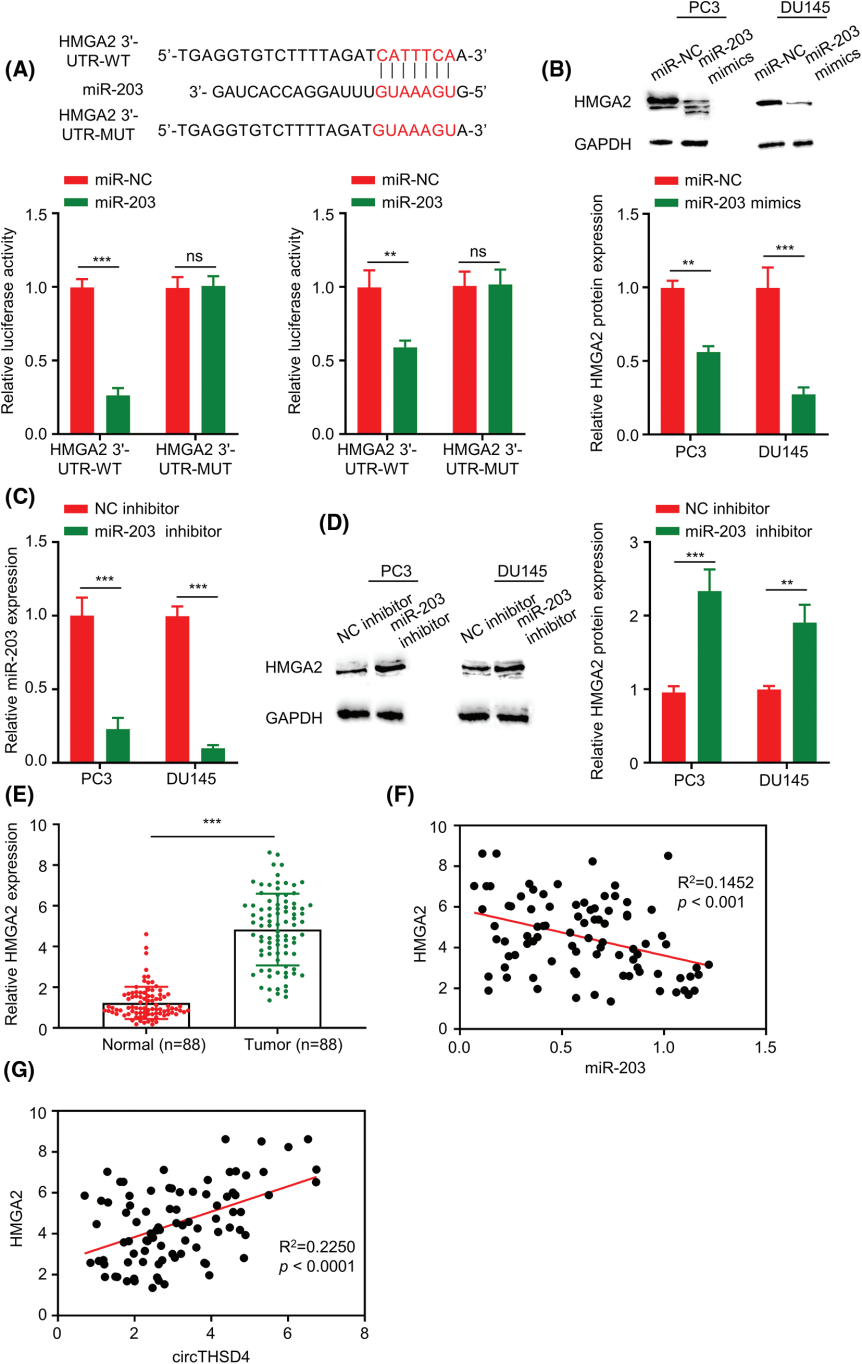
miR-203 negatively regulates HMGA2 level within PCa cells. (A) Estimated binding sequences (WT) and the mutated sequences (MUT) between miR-203 and 3′ untranslated region in HMGA2 mRNA. The interaction was validated through luciferase reporter assay within PC3 and DU145 cells. (B). WB analysis on HMGA2 protein expression within PC3 and DU145 cells upon transfection of miR-NC or miR-203 mimics. (C) RT-qPCR analysis of miR-203 levels within PC3 and DU145 cells upon the transfection of inhibitor-NC or miR-203 inhibitor. (D) WB assay on HMGA2 protein expression within PC3 and DU145 cells upon the transfection of inhibitor-NC or miR-203 inhibitor. (E) HMGA2 mRNA levels within the 88 pairs of PCa together with non-carcinoma samples detected by RT-qPCR assay. (F) Relation of HMGA2 with miR-203 levels within PCa samples. (G) Relation of circTHSD4 with HMGA2 levels within PCa. *p < 0.05, **p < 0.01, ***p < 0.001, and ****p < 0.0001.

TargetScan prediction was adopted for investigating the potential mRNA targets of miR-203. There were putative binding sequences of miR-203 at the 3′ UTR of HMGA2 mRNA. Further, the luciferase reporter assays demonstrated that overexpressing miR-203 in PC3 and DU145 cells hindered the activity of WT reporter, whereas the impact was not found in the MUT reporter (Fig. 5A). HMGA2 protein levels were redcued after overexpressing miR-203 (Fig. 5B). In contrast, when the expression of miR-203 was suppressed in PC3 and DU145 cells by miR-203 inhibitor (*p* < 0.001, Fig. 5C), HMGA2 protein levels were significantly increased (Fig. 5D). To this end, HMGA2 expression levels were also examined in 88 pairs of PCa tumors and para-cancerous tissues. HMGA2 mRNA levels were considerably higher in the PCa tumors in relative to the para-cancerous normal tissues (*p* < 0.001, Fig. 5E). Moreover, HMGA2 and miR-203 expression levels showed trend of an inverse correlation (R^2^ = 0.1452, *p* < 0.001, Fig. 5F) and a positive association between circTHSD4 and HMGA2 levels was observed (R^2^ = 0.2250, *p* < 0.0001, Fig. 5G).

### circTHSD4 controls the malignancy of PCa cells through targeting miR-203/HMGA2 axis

**Figure 6:**
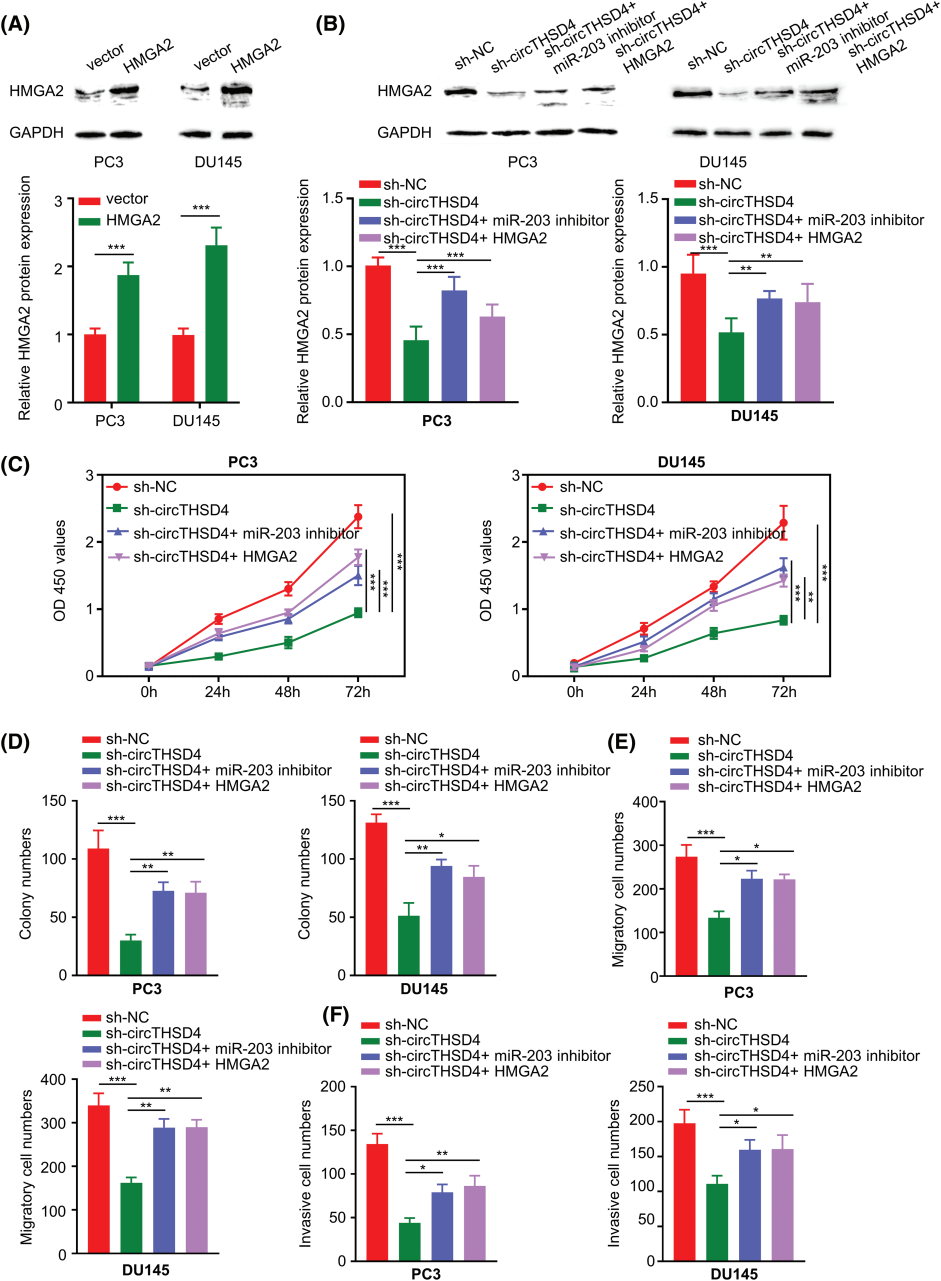
circTHSD4 modulates PCa cell growth, invasion and migration via miR-203/HMGA2 axis. (A and B) WB analyses on HMGA2 protein expression within PC3 and DU145 cells under specific experimental conditions. (C) CCK-8 viability test, (D) The colony formation, and (E and F) Transwell invasion and migration assays in PC3 and DU145 cells. *p < 0.05, **p < 0.01, ***p < 0.001, and ****p < 0.0001.

According to the above analysis, the function of circTHSD4 in PCa could be partially mediated by targeting the miR-203/HMGA2 axis. To explore this hypothesis, we constructed an HMGA2 expression vector to overexpress HMGA2 in PC3 and DU145 cells (*p* < 0.001, Fig. 6A). circTHSD4 knockdown decreased the HMGA2 protein levels, whereas the transfection of miR-203 inhibitor or HMGA2 expression vector could partially restore HMGA2 levels (*p* < 0.001, Fig. 6B). In the above experimental groups, the role of circTHSD4/miR-203/HMGA2 axis in dictating the malignancy of PCa cells was explored by different functional assays. The cell growth *t* was retarded upon the knockdown of circTHSD4. In contrast, the transfection with miR-203 inhibitor or HMGA2 expression plasmid partially enhanced the proliferation of PCa cells (*p* < 0.001, Fig. 6C). Besides, the suppressive effect of circTHSD4 knockdown on the colony formation was also partially rescued after the transfection with miR-203 inhibitor or HMGA2 expression vector (*p* < 0.001, Fig. 6D). miR-203 inhibition and HMGA2 expression could also enhance migratory and invasion abilities in PCa cells with circTHSD4 knockdown (Figs. 6E and 6F). Therefore, these data indicate that the functional role of circTHSD4 in PCa cells is at least partially mediated by the miR-203/HMGA2 axis.

### circTHSD4/miR-203/HMGA2 axis regulates the sensitivity of PCa cells to DTX

**Figure 7:**
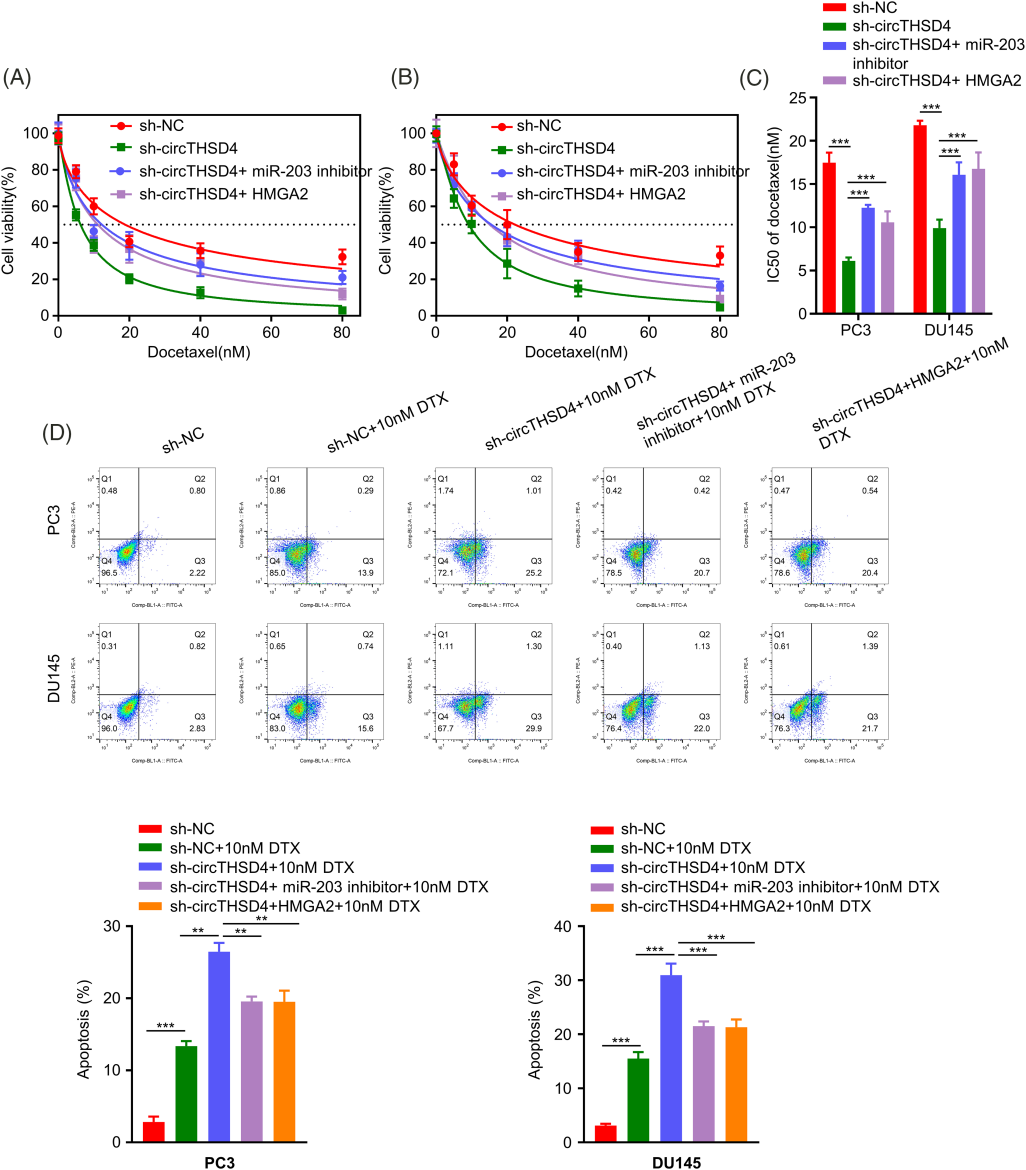
circTHSD4 regulates DTX sensitivity in PCa cells via miR-203/HMGA2 axis. (A and B) CCK-8 cell viability tests within PC3 and DU145 cells following treatment with DTX at various doses for 48 h. (C) The summary of IC50 values in PC3 and DU145 cells. (D) Flow cytometry on apoptosis cell proportion after DTX treatment in PC3 and DU145 cells. *p < 0.05, **p < 0.01, ***p < 0.001, and ****p < 0.0001.

We next investigated the engagement of circTHSD4/miR-203/HMGA2 axis in dictating the sensitivity of PCa cells to DTX treatment. PC3 and DU145 cells in different experimental groups were treated with DTX for 48 h and the cell viability was assessed by CCK-8 assay. Silencing circTHSD4 promoted the sensitivity of PCa cells to DTX treatment. The transfection of miR-203 inhibitor or an HMGA2 expression vector reduced DTX-induced toxicity in the sh-circTHSD4 group (Figs. 7A–7C). In addition, apoptosis detection by flow cytometry also demonstrated that the proportion of apoptotic events in DTX-treated cells was increased upon circTHSD4 silencing, and the simultaneous transfection with miR-203 inhibitor or HMGA2 expression vector decreased the level of apoptosis upon circTHSD4 silencing (Fig. 7D).

## Discussion

The current study revealed the important roles and the molecular mechanisms of circTHSD4 in modulating the progression and drug sensitivity of DTX in PCa. The elevated expression levels of circTHSD4 are associated with the poor prognosis in PCa patients. A high level of circTHSD4 expression was also strongly correlated to the tumor stage, lymph node metastasis, and Gleason score. It was further shown that circTHSD4 might act as a sponge for miR-203, which limits its suppression on the downstream target mRNA of HMGA2. circTHSD4/miR-203/HMGA2 axis not only regulates the malignant phenotype of PCa cells, but also controls the sensitivity of PCa cells to DTX. Thus, our data highlight the potential role of circTHSD4/miR-203/HMGA2 axis in the malignant progression of PCa.

circRNAs are generated by the back-splicing of exons or introns of protein-coding genes, which can be classified into three categories on the basis of the origin (ecircRNAs, eicircRNAs, and icirRNAs) [26]. For example, circTHSD4 is an ecircRNA composed of two exons from the THSD4 coding sequences. The deregulation of circRNAs has a significant implication in tumorigenesis. Gao et al. [27] reported that hsa_circ_0000735 was abundantly expressed in DTX-resistant PCa tissues when compared to DTX-sensitive PCa tissues, and mechanistic analysis revealed that hsa_circ_0000735 functions as a molecular sponge for miR-7-5p. In our study, we observed an increased sensitivity to DTX in PC3 and DU145 cell lines after circTHSD4 knockdown. The enhanced DTX sensitivity by circTHSD4 silencing could be partially reversed by lowering miR-203 expression or overexpressing HMGA2. Recent evidence suggests that circRNAs were enriched with miRNA response elements (MREs) to adsorb miRNAs and modulate their activities [28]. In addition, miRNAs regulate biological processes by binding to the complementary sites within the 3′-UTR of mRNAs and repressing the levels of target mRNAs [29]. By controlling the expression of pro- or oncogenes, miRNAs can impact on tumor cell growth, invasion, and colony formation [30]. Through absorbing miRNAs, circRNAs act as the upstream regulators to control the expression of miRNA targets. Importantly, compared to other miRNA sponges, some circRNAs can be considered as “super sponges” which possess binding sequences to adsorb multiple miRNAs [31]. For instance, bioinformatics prediction indicates that circTHSD4 can potentially interact with different miRNAs. However, we observed that miR-203 was mostly enriched by the biotinylated circTHSD4 probe in RNA pull-down assay, suggesting that circTHSD4 might play the role of a sponge for miR-203. The elevated circTHSD4 expression in PCa tumor tissues was correlated with the reduced miR-203 level in PCa tumor tissues. In addition, we demonstrated the cytoplasmic location of circTHSD4, indicating that circTHSD4 may predominantly function in the cytoplasm.

A previous study indicates that miR-203 was located on chromosome 14q32.33 [32], which may target 588 genes in human genome and is implicated in the pathological processes of several diseases [33,34]. Previous studies demonstrate that miR-203 serves as a tumor suppressor in PCa by binding to various mRNA targets. For instance, the proliferation of PCa cells could be hindered by miR-203 through targeting IRS-1 [35]. By downregulating Rap1A expression, miR-203 was discovered to suppress the cell growth, migration, and invasion abilities in PCa cells [36]. In another instance, the reduced expression of miR-203 was also associated with the acquisition of tyrosine kinase inhibitor resistance in PCa [37]. In our work, we revealed that circTHSD4 acts as an upstream regulator of miR-203 and HMGA2. Silencing circTHSD4 suppressed the proliferation and the tumor formation of PCa cells through targeting miR-203/HMGA2 axis. circTHSD4 knockdown also promoted DTX sensitivity in PCa cells. Thus, our data uncover a novel mechanism by which miR-203 serves as a regulatory target of circTHSD4 in dictating the malignancy and DTX drug resistance of PCa cells.

HMGA2 is a vital epigenetic regulator engaged in organism development, cell differentiation and pathophysiological conditions. Although barely detectable in differentiated tissues, HMGA2 is highly expressed in embryonic tissues and malignant tumors (18–20). Rats with defective HMGA2 gene exhibit a dwarf phenotype, suggesting a crucial role of HMGA2 in the organ development and tissue growth [38]. The overexpression of HMGA2 was widely reported in malignant tumors. For instance, a meta-analysis of 19 different malignancies suggested that the expression of HMGA2 presented upregulation in most cancer types, and HMGA2 overexpression was associated with vascular invasion, lymphatic metastasis, and poor prognosis [39]. HMGA2 functions to promote the epithelial-mesenchymal transition in PCa cells through activating the mitogen‑activated protein kinase (MAPK) pathway [40]. Further, HMGA2 silencing resulted in apoptosis of tumor cells and impaired the migration and invasion abilities (40). In our study, we showed that circTHSD4/miR-203 axis regulates the protein level of HMGA2. HMGA2 overexpression could partially rescue the the proliferation, migration, as well as invasion abilities of PCa cells upon circTHSD4 knockdown. HMGA2 overexpression also promoted DTX drug resistance in PCa cells. Therefore, these findings are in agreement with the tumor-promoting effect of HMGA2, but also pinpoint the circTHSD4/miR-203 axis as a regulatory module for HMGA2 expression in PCa cells.

However, since circRNAs may also interact directly with protein partners to regulate biological processes, we could not exclude the possibility that circTHSD4 might bind to protein targets to modulate the malignancy and DTX drug sensitivity in PCa cells. In addition, circRNAs may also target other non-coding RNAs in PCa cells. Therefore, future work is required to systematically profile the RNA and protein targets of circTHSD4 using pull-down and omics approaches. In addition, considering that the correlation trends of circTHSD4, miR-203 and HMGA2 were relatively weak in PCa tumor samples, a larger size of clinical samples could provide more convincing conclusion regarding the expression pattern of these molecules in PCa cells.

## Conclusion

To conclude, our study indicated that circTHSD4 expression was elevated in the PCa tissues and cells, and the heightened expression of circTHSD4 was linked with a poor prognosis in PCa patients. The overexpression of circTHSD4 is not only needed to support the malignancy of PCa cells, but also promotes the resistance to DTX drug resistance. circTHSD4 modulates the expression of HMGA2 indirectly by sponging miR-203. HMGA2 overexpression may foster the tumor progression and DTX resistance in the PCa cells. The obtained findings imply that the high level expression of circTHSD4 may contribute to the development of DTX resistance in PCa patients, and targeting circTHSD4 is a potential strategy to enhance the DTX treatment efficacy in PCa management.

## Supplementary Materials

**Table S1:** Sequences of primers used for RT-qPCR analysis

Primer name		Sequence (from 5′ to 3′)	Tm value
circTHSD4	Forward	TTCATGGGGTCTCTCAGTGTC	56.9°C
	Reverse	AGACGTTGCCAATTTTCTGC	57.9°C
HMGA2	Forward	ACCCAGGGGAAGACCCAAA	61.2°C
	Reverse	CCTCTTGGCCGTTTTTCTCCA	64.0°C
GAPDH	Forward	CTGGGCTACACTGAGCACC	55.9°C
	Reverse	AAGTGGTCGTTGAGGGCAATG	62.2°C
U6	Forward	ATTGGAACGATACAGAGAAGATT	54.7°C
	Reverse	GGAACGCTTCACGAATTTG	56.4°C
miR-203	Forward	GCAGGATCACCAGGATTTGT	57.0°C
	Reverse	GGTCCAGTTTTTTTTTTTTTTTCACT	61.5°C

## Data Availability

The data can be obtained from the corresponding author upon reasonable request.
